# The crystal structure of 1,5-dibenzyl-1*H*-pyrazolo­[3,4-*d*]pyrimidine-4(5*H*)-thione

**DOI:** 10.1107/S205698901402828X

**Published:** 2015-01-10

**Authors:** Mohammed El Fal, Youssef Ramli, El Mokhtar Essassi, Mohamed Saadi, Lahcen El Ammari

**Affiliations:** a Laboratoire de Chimie Organique Hétérocyclique URAC 21, Pôle de Compétence Pharmacochimie, Av. Ibn Battouta, BP 1014, Faculté des Sciences, Université Mohammed V, Rabat, Morocco; b Medicinal Chemistry Laboratory, Faculty of Medicine and Pharmacy, Mohammed V University, Rabat, Morocco; c Laboratoire de Chimie du Solide Appliquée, Faculté des Sciences, Université Mohammed V, Avenue Ibn Battouta, BP 1014, Rabat, Morocco

**Keywords:** crystal structure, pyrazolo­[3,4-*d*]pyrimidine, thione, C—H⋯S inter­actions

## Abstract

In the title compound, C_19_H_16_N_4_S, the pyrazolo­[3,4-*d*]pyrimidine ring is close to being planar, with the greatest deviation from the mean plane being 0.023 (2) Å for the C atom bearing the thione S atom. The two phenyl rings are nearly perpendicular to the fused ring system [dihedral angles = 71.4 (2) and 78.1 (2)°], but are oriented in opposite directions; the dihedral angle between the phenyl rings is 32.22 (16)°. In the crystal, linear supra­molecular chains along [101] are sustained by C—H⋯S inter­actions.

## Related literature   

For pharmacological and biochemical properties of pyrazolo­[1,5-*a*]pyrimidine, see: Orlikova *et al.* (2014 ▸); Yuan *et al.* (2013 ▸); Rashad *et al.* (2011 ▸). For related structures, see: El Fal *et al.* (2013 ▸, 2014 ▸); Alsubari *et al.* (2011 ▸); Ramli *et al.* (2012 ▸).
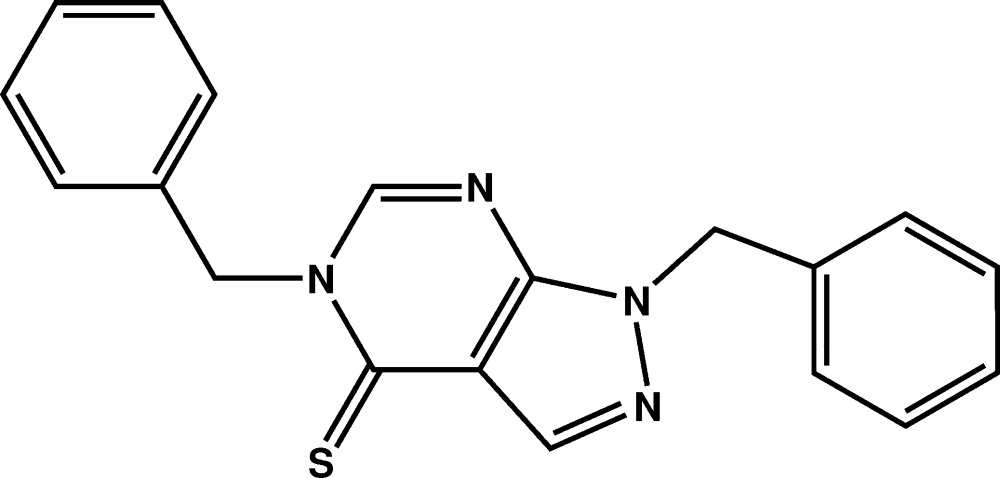


## Experimental   

### Crystal data  

C_19_H_16_N_4_S
*M* *_r_* = 332.42
Monoclinic, 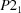

*a* = 4.4953 (12) Å
*b* = 29.140 (8) Å
*c* = 6.3889 (16) Å
β = 97.860 (9)°
*V* = 829.0 (4) Å^3^
*Z* = 2
Mo *K*α radiation
μ = 0.20 mm^−1^
*T* = 296 K
0.37 × 0.34 × 0.29 mm

### Data collection  

Bruker X8 APEX diffractometer
Absorption correction: multi-scan (*SADABS*; Bruker, 2009 ▸) *T*_min_ = 0.589, *T*_max_ = 0.746
9214 measured reflections
3582 independent reflections
2406 reflections with *I* > 2σ(*I*)
*R*_int_ = 0.040

### Refinement  

*R*[*F* ^2^ > 2σ(*F* ^2^)] = 0.041
*wR*(*F* ^2^) = 0.084
*S* = 0.97
3582 reflections
217 parameters
1 restraint
H-atom parameters constrained
Δρ_max_ = 0.14 e Å^−3^
Δρ_min_ = −0.12 e Å^−3^
Absolute structure: Flack & Bernardinelli (2000 ▸), 1730 Friedel pairs
Absolute structure parameter: −0.11 (7)

### 

Data collection: *APEX2* (Bruker, 2009 ▸); cell refinement: *SAINT-Plus* (Bruker, 2009 ▸); data reduction: *SAINT-Plus*; program(s) used to solve structure: *SHELXS97* (Sheldrick, 2008 ▸); program(s) used to refine structure: *SHELXL97* (Sheldrick, 2008 ▸); molecular graphics: *ORTEP-3 for Windows* (Farrugia, 2012 ▸); software used to prepare material for publication: *PLATON* (Spek, 2009 ▸) and *publCIF* (Westrip, 2010 ▸).

## Supplementary Material

Crystal structure: contains datablock(s) I. DOI: 10.1107/S205698901402828X/tk5354sup1.cif

Structure factors: contains datablock(s) I. DOI: 10.1107/S205698901402828X/tk5354Isup2.hkl

Supporting information file. DOI: 10.1107/S205698901402828X/tk5354Isup3.cml

**Fig. 1.**
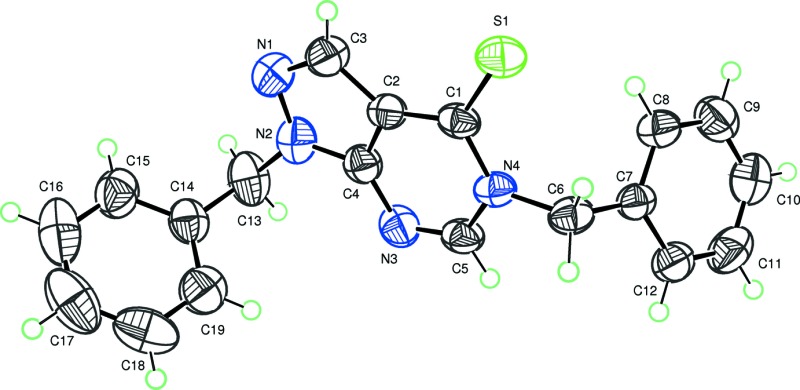
Molecular structure of the title compound with the atom-labelling scheme. Displacement ellipsoids are drawn at the 50% probability level. H atoms are represented as small circles.

. DOI: 10.1107/S205698901402828X/tk5354fig1.tif
Mol­ecular structure of the title compound with the atom-labelling scheme. Displacement ellipsoids are drawn at the 50% probability level. H atoms are represented as small circles.

CCDC reference: 1041681

Additional supporting information:  crystallographic information; 3D view; checkCIF report

## Figures and Tables

**Table 1:** Hydrogen-bond geometry (, )

*D*H*A*	*D*H	H*A*	*D* *A*	*D*H*A*
C5H5S1^i^	0.93	2.87	3.784(3)	167

Symmetry code: (i) 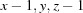.

## supplementary crystallographic information

### S1. Structural commentary

Pyrazolo­[3,4-d] pyrimidine-4-thione are inter­mediate sub-units useful for the development of molecules of pharmaceutical inter­est. They have found applications in various therapeutic areas, including anti-inflammatory, anti-tumour and anti-cancer (Orlikova *et al.*, 2014; Yuan *et al.*, 2013; Rashad *et al.*, 2011). The present paper is a continuation of our research work devoted to the development of pyrazolo­[3,4-*d*] pyrimidine derivatives with potential pharmacological activities (El Fal *et al.*, 2013; El Fal *et al.*, 2014; Alsubari *et al.*, 2011; Ramli *et al.*, 2012).

The molecule of the title compound is build up from two fused five- and six-membered heterocycles linked to two phenyl rings *via* two –CH_2_– groups as shown in Fig. 1. The pyrazolo­[3,4-*d*]pyrimidine system is virtually planar with the largest deviation from the mean plane being -0.023 (2) Å at C1 and makes dihedral angles of 71.4 (2)° and 78.1 (2)° with the mean plane through the first (C7 to C12) and the second (C14 to C19) phenyl rings, respectively. As a matter of fact, the two phenyl rings are oriented in opposite direction to the plane of the fused rings. No classic hydrogen bonds are observed in the present structure.

### S2. Synthesis and crystallization

3.32 g (10 mmol) of 1,5-di­benzyl-1H, 4H, 5*H*-pyrazolo [3,4-d] pyrimidin-4-one is refluxed in pyridine (30 ml) with 5.55 g (25 mmol) of phospho­rus penta­sulfide for 4 h. Then the solvent was evaporated under reduced pressure. The precipitate that formed was washed with hot water to remove residual dimerized P_2_S_5_ until colourless filtrate was noted. The solid was re-crystallized from ethanol to afford the title compound as yellow crystals (yield: 85%; m.p. = 563 K).

### S3. Refinement

The H atoms were located in a difference map and treated as riding with C—H = 0.93 Å (aromatic) and C—H = 0.97 Å (methyl­ene). All hydrogen with *U*_iso_(H) = 1.2 *U*_eq_ (aromatic and methyl­ene). Two reflections, *i.e*. 0 -2 0 and 0 2 0, were omitted fro the final refinement owing to poor agreement.

### Crystal data

**Table:**

C_19_H_16_N_4_S	*F*(000) = 348
*M**_r_* = 332.42	*D*_x_ = 1.332 Mg m^−^^3^
Monoclinic, *P*2_1_	Melting point: 563 K
Hall symbol: P 2yb	Mo *K*α radiation, λ = 0.71073 Å
*a* = 4.4953 (12) Å	Cell parameters from 3582 reflections
*b* = 29.140 (8) Å	θ = 2.8–27.1°
*c* = 6.3889 (16) Å	µ = 0.20 mm^−^^1^
β = 97.860 (9)°	*T* = 296 K
*V* = 829.0 (4) Å^3^	Block, yellow
*Z* = 2	0.37 × 0.34 × 0.29 mm

### Data collection

**Table:**

Bruker X8 APEX diffractometer	3582 independent reflections
Radiation source: fine-focus sealed tube	2406 reflections with *I* > 2σ(*I*)
Graphite monochromator	*R*_int_ = 0.040
φ and ω scans	θ_max_ = 27.1°, θ_min_ = 2.8°
Absorption correction: multi-scan (*SADABS*; Bruker, 2009)	*h* = −5→5
*T*_min_ = 0.589, *T*_max_ = 0.746	*k* = −37→37
9214 measured reflections	*l* = −8→8

### Refinement

**Table:**

Refinement on *F*^2^	Secondary atom site location: difference Fourier map
Least-squares matrix: full	Hydrogen site location: inferred from neighbouring sites
*R*[*F*^2^ > 2σ(*F*^2^)] = 0.041	H-atom parameters constrained
*wR*(*F*^2^) = 0.084	*w* = 1/[σ^2^(*F*_o_^2^) + (0.0318*P*)^2^] where *P* = (*F*_o_^2^ + 2*F*_c_^2^)/3
*S* = 0.97	(Δ/σ)_max_ < 0.001
3582 reflections	Δρ_max_ = 0.14 e Å^−^^3^
217 parameters	Δρ_min_ = −0.12 e Å^−^^3^
1 restraint	Absolute structure: Flack & Bernardinelli (2000), 1730 Friedel pairs
Primary atom site location: structure-invariant direct methods	Absolute structure parameter: −0.11 (7)

### Special details

**Table:**

Geometry. All e.s.d.'s (except the e.s.d. in the dihedral angle between two l.s. planes) are estimated using the full covariance matrix. The cell e.s.d.'s are taken into account individually in the estimation of e.s.d.'s in distances, angles and torsion angles; correlations between e.s.d.'s in cell parameters are only used when they are defined by crystal symmetry. An approximate (isotropic) treatment of cell e.s.d.'s is used for estimating e.s.d.'s involving l.s. planes.
Refinement. Refinement of *F*^2^ against all reflections. The weighted *R*-factor *wR* and goodness of fit *S* are based on *F*^2^, conventional *R*-factors *R* are based on *F*, with *F* set to zero for negative *F*^2^. The threshold expression of *F*^2^ > σ(*F*^2^) is used only for calculating *R*-factors(gt) *etc*. and is not relevant to the choice of reflections for refinement. *R*-factors based on *F*^2^ are statistically about twice as large as those based on *F*, and *R*- factors based on all data will be even larger.

### Fractional atomic coordinates and isotropic or equivalent isotropic displacement parameters (Å^2^)

**Table:**

	*x*	*y*	*z*	*U*_iso_*/*U*_eq_	
C1	0.5941 (5)	0.48633 (8)	0.9980 (3)	0.0412 (6)	
C2	0.4720 (5)	0.44502 (8)	1.0570 (3)	0.0411 (6)	
C3	0.5145 (7)	0.41623 (9)	1.2377 (4)	0.0586 (7)	
H3	0.6506	0.4222	1.3578	0.070*	
C4	0.2489 (6)	0.42310 (9)	0.9208 (4)	0.0457 (6)	
C5	0.2551 (6)	0.47501 (9)	0.6716 (4)	0.0488 (6)	
H5	0.1885	0.4864	0.5375	0.059*	
C6	0.5892 (5)	0.54088 (9)	0.6906 (4)	0.0477 (6)	
H6A	0.6197	0.5330	0.5476	0.057*	
H6B	0.7826	0.5495	0.7672	0.057*	
C7	0.3812 (5)	0.58142 (8)	0.6832 (4)	0.0425 (6)	
C8	0.3274 (6)	0.60388 (9)	0.8650 (4)	0.0545 (7)	
H8	0.4188	0.5935	0.9959	0.065*	
C9	0.1404 (7)	0.64137 (10)	0.8546 (5)	0.0651 (8)	
H9	0.1070	0.6563	0.9780	0.078*	
C10	0.0034 (7)	0.65686 (11)	0.6635 (5)	0.0700 (8)	
H10	−0.1242	0.6821	0.6569	0.084*	
C11	0.0546 (7)	0.63514 (11)	0.4817 (5)	0.0701 (9)	
H11	−0.0375	0.6458	0.3515	0.084*	
C12	0.2421 (6)	0.59761 (9)	0.4912 (4)	0.0551 (7)	
H12	0.2754	0.5830	0.3671	0.066*	
C13	−0.0331 (7)	0.34855 (9)	0.9311 (5)	0.0701 (9)	
H13A	−0.1746	0.3609	0.8167	0.084*	
H13B	−0.1461	0.3374	1.0396	0.084*	
C14	0.1353 (6)	0.30957 (9)	0.8500 (5)	0.0538 (7)	
C15	0.1948 (7)	0.27009 (11)	0.9677 (5)	0.0701 (8)	
H15	0.1235	0.2673	1.0970	0.084*	
C16	0.3567 (8)	0.23521 (11)	0.8969 (8)	0.0926 (12)	
H16	0.3946	0.2088	0.9778	0.111*	
C17	0.4635 (8)	0.23889 (15)	0.7078 (9)	0.0961 (13)	
H17	0.5758	0.2152	0.6605	0.115*	
C18	0.4049 (8)	0.27764 (16)	0.5874 (6)	0.0898 (11)	
H18	0.4766	0.2801	0.4581	0.108*	
C19	0.2406 (7)	0.31279 (11)	0.6575 (5)	0.0705 (8)	
H19	0.2000	0.3389	0.5748	0.085*	
N1	0.3381 (6)	0.38019 (8)	1.2147 (4)	0.0633 (6)	
N2	0.1709 (5)	0.38498 (7)	1.0188 (4)	0.0575 (6)	
N3	0.1303 (5)	0.43742 (7)	0.7249 (3)	0.0529 (5)	
N4	0.4758 (4)	0.49978 (6)	0.7928 (3)	0.0422 (5)	
S1	0.85051 (15)	0.51743 (3)	1.14918 (10)	0.0571 (2)	

### Atomic displacement parameters (Å^2^)

**Table:**

	*U*^11^	*U*^22^	*U*^33^	*U*^12^	*U*^13^	*U*^23^
C1	0.0382 (13)	0.0485 (15)	0.0369 (13)	0.0091 (11)	0.0049 (10)	−0.0077 (11)
C2	0.0406 (14)	0.0454 (15)	0.0366 (13)	0.0074 (11)	0.0030 (11)	−0.0022 (11)
C3	0.0693 (19)	0.0591 (18)	0.0468 (16)	0.0095 (16)	0.0061 (14)	0.0028 (14)
C4	0.0446 (15)	0.0458 (16)	0.0474 (15)	0.0080 (12)	0.0087 (13)	−0.0024 (13)
C5	0.0507 (16)	0.0561 (18)	0.0367 (13)	0.0104 (14)	−0.0046 (11)	−0.0056 (12)
C6	0.0459 (15)	0.0593 (16)	0.0395 (14)	−0.0032 (13)	0.0116 (12)	0.0003 (12)
C7	0.0424 (15)	0.0473 (14)	0.0375 (14)	−0.0095 (12)	0.0049 (11)	0.0009 (12)
C8	0.0618 (18)	0.0591 (18)	0.0412 (15)	−0.0018 (14)	0.0023 (13)	0.0010 (13)
C9	0.071 (2)	0.0571 (19)	0.069 (2)	−0.0002 (16)	0.0151 (17)	−0.0094 (15)
C10	0.071 (2)	0.0573 (19)	0.082 (2)	0.0038 (16)	0.0097 (17)	0.0114 (18)
C11	0.073 (2)	0.073 (2)	0.0607 (19)	0.0017 (18)	−0.0028 (16)	0.0207 (17)
C12	0.0593 (18)	0.0584 (18)	0.0467 (15)	−0.0083 (15)	0.0040 (13)	0.0037 (13)
C13	0.0531 (18)	0.0551 (19)	0.102 (2)	−0.0104 (15)	0.0102 (17)	−0.0024 (16)
C14	0.0479 (16)	0.0475 (16)	0.0647 (18)	−0.0101 (12)	0.0028 (14)	−0.0036 (14)
C15	0.074 (2)	0.0588 (19)	0.076 (2)	−0.0095 (17)	0.0032 (16)	0.0059 (17)
C16	0.079 (3)	0.053 (2)	0.140 (4)	0.0019 (19)	−0.006 (3)	0.003 (2)
C17	0.062 (2)	0.080 (3)	0.144 (4)	0.000 (2)	0.004 (3)	−0.041 (3)
C18	0.076 (3)	0.116 (3)	0.077 (2)	−0.014 (2)	0.0093 (19)	−0.036 (3)
C19	0.069 (2)	0.069 (2)	0.072 (2)	−0.0072 (16)	0.0068 (17)	−0.0002 (16)
N1	0.0782 (18)	0.0568 (16)	0.0564 (15)	−0.0011 (13)	0.0142 (13)	0.0070 (12)
N2	0.0550 (15)	0.0504 (14)	0.0681 (16)	0.0002 (11)	0.0118 (12)	0.0033 (13)
N3	0.0503 (13)	0.0496 (14)	0.0558 (14)	0.0029 (11)	−0.0034 (11)	−0.0043 (11)
N4	0.0406 (11)	0.0509 (12)	0.0342 (10)	0.0034 (9)	0.0021 (9)	−0.0013 (9)
S1	0.0536 (4)	0.0676 (4)	0.0463 (4)	−0.0042 (4)	−0.0073 (3)	−0.0052 (4)

### Geometric parameters (Å, º)

**Table:**

C1—C2	1.396 (3)	C10—C11	1.370 (4)
C1—N4	1.402 (3)	C10—H10	0.9300
C1—S1	1.667 (2)	C11—C12	1.377 (4)
C2—C4	1.390 (3)	C11—H11	0.9300
C2—C3	1.419 (3)	C12—H12	0.9300
C3—N1	1.312 (3)	C13—N2	1.463 (3)
C3—H3	0.9300	C13—C14	1.496 (4)
C4—N2	1.345 (3)	C13—H13A	0.9700
C4—N3	1.357 (3)	C13—H13B	0.9700
C5—N3	1.297 (3)	C14—C19	1.380 (4)
C5—N4	1.376 (3)	C14—C15	1.380 (4)
C5—H5	0.9300	C15—C16	1.363 (5)
C6—N4	1.487 (3)	C15—H15	0.9300
C6—C7	1.503 (3)	C16—C17	1.364 (5)
C6—H6A	0.9700	C16—H16	0.9300
C6—H6B	0.9700	C17—C18	1.371 (5)
C7—C12	1.381 (3)	C17—H17	0.9300
C7—C8	1.383 (3)	C18—C19	1.373 (5)
C8—C9	1.374 (4)	C18—H18	0.9300
C8—H8	0.9300	C19—H19	0.9300
C9—C10	1.367 (4)	N1—N2	1.376 (3)
C9—H9	0.9300		
			
C2—C1—N4	112.4 (2)	C11—C12—C7	120.7 (3)
C2—C1—S1	125.35 (18)	C11—C12—H12	119.6
N4—C1—S1	122.28 (18)	C7—C12—H12	119.6
C4—C2—C1	120.3 (2)	N2—C13—C14	111.3 (2)
C4—C2—C3	104.1 (2)	N2—C13—H13A	109.4
C1—C2—C3	135.7 (2)	C14—C13—H13A	109.4
N1—C3—C2	111.7 (3)	N2—C13—H13B	109.4
N1—C3—H3	124.1	C14—C13—H13B	109.4
C2—C3—H3	124.1	H13A—C13—H13B	108.0
N2—C4—N3	126.1 (2)	C19—C14—C15	118.5 (3)
N2—C4—C2	107.4 (2)	C19—C14—C13	120.5 (3)
N3—C4—C2	126.5 (2)	C15—C14—C13	120.9 (3)
N3—C5—N4	126.9 (2)	C16—C15—C14	120.9 (3)
N3—C5—H5	116.6	C16—C15—H15	119.6
N4—C5—H5	116.6	C14—C15—H15	119.6
N4—C6—C7	113.42 (17)	C15—C16—C17	120.3 (4)
N4—C6—H6A	108.9	C15—C16—H16	119.9
C7—C6—H6A	108.9	C17—C16—H16	119.9
N4—C6—H6B	108.9	C16—C17—C18	119.8 (4)
C7—C6—H6B	108.9	C16—C17—H17	120.1
H6A—C6—H6B	107.7	C18—C17—H17	120.1
C12—C7—C8	118.3 (2)	C17—C18—C19	120.1 (4)
C12—C7—C6	120.0 (2)	C17—C18—H18	119.9
C8—C7—C6	121.7 (2)	C19—C18—H18	119.9
C9—C8—C7	120.7 (3)	C18—C19—C14	120.3 (3)
C9—C8—H8	119.6	C18—C19—H19	119.8
C7—C8—H8	119.6	C14—C19—H19	119.8
C10—C9—C8	120.3 (3)	C3—N1—N2	105.5 (2)
C10—C9—H9	119.9	C4—N2—N1	111.2 (2)
C8—C9—H9	119.9	C4—N2—C13	127.6 (2)
C9—C10—C11	119.8 (3)	N1—N2—C13	120.7 (2)
C9—C10—H10	120.1	C5—N3—C4	111.8 (2)
C11—C10—H10	120.1	C5—N4—C1	122.1 (2)
C10—C11—C12	120.1 (3)	C5—N4—C6	116.1 (2)
C10—C11—H11	119.9	C1—N4—C6	121.78 (19)
C12—C11—H11	119.9		

### Hydrogen-bond geometry (Å, º)

**Table:**

*D*—H···*A*	*D*—H	H···*A*	*D*···*A*	*D*—H···*A*
C5—H5···S1^i^	0.93	2.87	3.784 (3)	167

Symmetry code: (i) *x*−1, *y*, *z*−1.
