# Supplementary figures and images for: The crystal structure of 1,5-dibenzyl-1H-pyrazolo­[3,4-d]pyrimidine-4(5H)-thione
Source: Acta Crystallogr E Crystallogr Commun. 2015 Jan 10;71(Pt 2):o95–6. doi: 10.1107/S205698901402828X (PMC4384557; doi:10.1107/S205698901402828X)

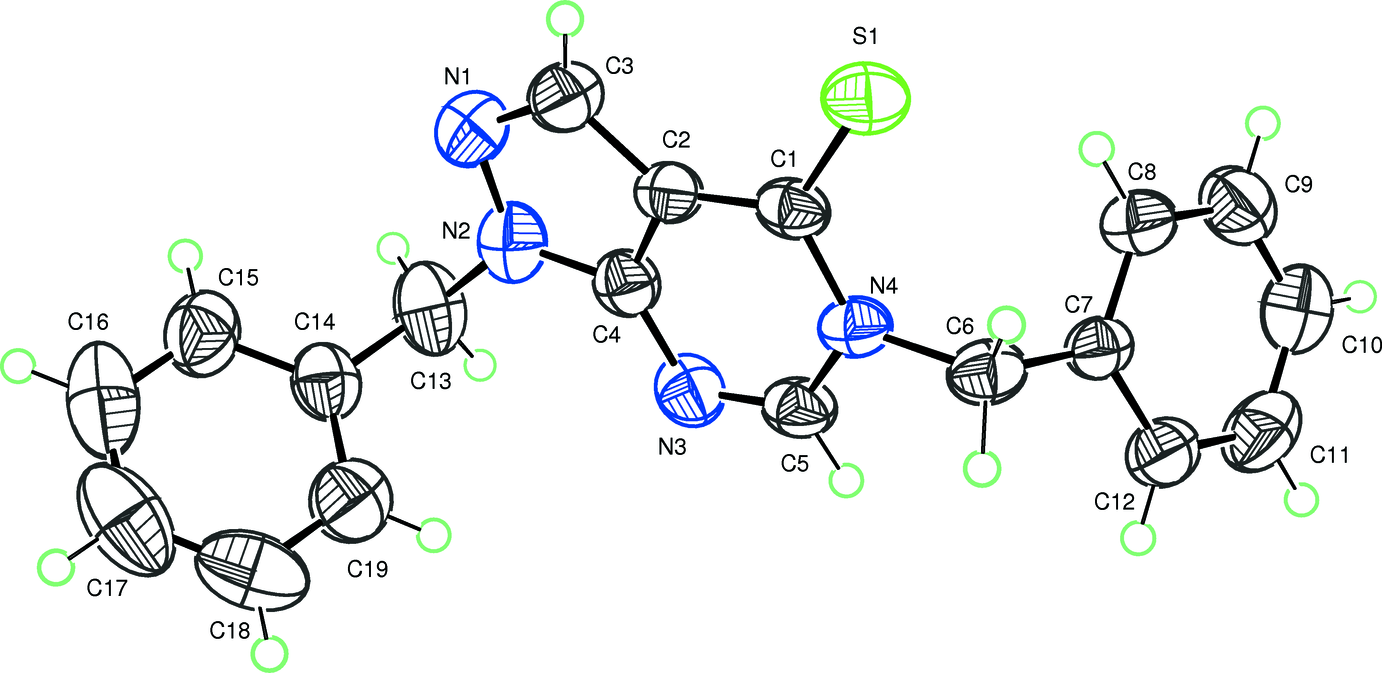

Supplement: Supplementary file 4 [file e-71-00o95-fig1.tif]
